# Supplementary material for: A Pilot Study Evaluating LV Diastolic Function with M-Mode Measurement of Mitral Valve Movement in the Parasternal Long Axis View
Source: Diagnostics (Basel). 2023 Jul 19;13(14):2412. doi: 10.3390/diagnostics13142412 (PMC10378499; doi:10.3390/diagnostics13142412)
Supplement: Supplementary file 1 [file diagnostics-13-02412-s001.zip › diagnostics-2433129-supplementary.pdf]

Supplementary Material

Table S1. Participants demographics

N = 30	Mean ± SD, Median [IQR]
Male (%)	20 (66.7)
Female (%)	10 (33.3)
Age (year)	28 [26 - 30]
Body mass index	22.7 ± 2.3
Height (cm)	169.7 ± 6.9
Weight (kg)	65.7 ± 10.3
SBP (mmHg)	124.0 ± 9.4
DBP (mmHg)	78.2 ± 8.5
HR (/min)	82.0 ± 13.2

Continuous variables with normal distribution are presented as mean ± standard deviation (SD). Continuous variables with non-normal distribution are presented as median [IQR, interquartile range]
